# How Does Social Security Fairness Predict Trust in Government? The Serial Mediation Effects of Social Security Satisfaction and Life Satisfaction

**DOI:** 10.3390/ijerph19116867

**Published:** 2022-06-03

**Authors:** Kuiyun Zhi, Qiurong Tan, Si Chen, Yongjin Chen, Xiaoqin Wu, Chenkai Xue, Anbang Song

**Affiliations:** 1 School of Public Policy and Administration, Chongqing University, Chongqing 400044, China; kyzhi@cqu.edu.cn (K.Z.); tanqr@cqu.edu.cn (Q.T.); wuxq@cqu.edu.cn (X.W.); xck@cqu.edu.cn (C.X.); 202101021025@cqu.edu.cn (A.S.); 2 School of Business Administration, Chongqing Technology and Business University, Chongqing 400067, China; chensi1991@ctbu.edu.cn

**Keywords:** trust in government, social security fairness, social security satisfaction, life satisfaction

## Abstract

Several studies have found that trust in government is associated with social fairness, citizens’ satisfaction with public service, and life satisfaction. This study aimed to investigate the serial mediation effects of social security satisfaction and life satisfaction on the association between social security fairness and trust in government. We analyzed the data from the Chinese Social Survey in 2019 (*n* = 7403) to examine the serial mediation effects. The findings showed that the higher the level of government, the greater the trust it enjoyed from its citizens. The direct prediction of trust by social security fairness was stronger at the county and township levels than at the central government level. Both social security satisfaction and life satisfaction partially mediated the relationship between social security fairness and overall trust in government. Social security fairness indirectly positively predicted trust in local government at the county and township levels through social security satisfaction, life satisfaction, and their serial mediation. While social security fairness could only indirectly predict trust in central government through social security satisfaction, the prediction of trust in central government via life satisfaction (mediator) was not significant. We observed a serial mediation model in which social security fairness positively predicted trust in government directly and indirectly through social security satisfaction and life satisfaction. The finding that social security satisfaction partially mediates the relationship between perceptions of fairness in the social security system and trust in government has implications for improving policies and the functioning of the system at all levels of the government.

## 1. Introduction

With the spread of COVID-19, in many countries, including China, citizens’ trust in government has become more important [1]. Trust in government refers to the citizens’ belief or confidence that the government will produce results consistent with their expectations [2,3], which is the core foundation of effective governance [1]. Extensive studies have been conducted to explore the factors of trust in government, including government performance [4,5], fairness [6,7], public service [8], and citizen satisfaction [9,10]. Citizens’ requirements of the government have gradually changed from economic development to livelihood issues such as public services and social fairness in the COVID-19 era [11]. The importance of social fairness and public services has exceeded that of economic performance in mediating trust in government [11].

As an important element of basic public services, fairness is the core concept and primary principle of social security [12]. The issue of fairness in China’s social security still exists [12]. The government provides social security [13]; thus, if citizens feel that the social security system is unfair and that the government’s management of social security is inconsistent with citizens’ expectations, they may lose trust in the government [14]. Therefore, it seems reasonable to assume that social security fairness is related to trust in government.

On the one hand, fairness is one of the standards that citizens use to evaluate the quality of social security, which may affect their satisfaction with social security [15,16]. On the other hand, citizens’ daily life is closely related to social security, whereby its improved supply level and fairness could significantly promote citizens’ life satisfaction [13]. Previous studies have indicated that social security satisfaction and life satisfaction are positively associated with citizens’ trust in government [17,18]. Thus, it can be seen that social security fairness, social security satisfaction, life satisfaction, and trust in government are closely related.

However, we know little about how social security fairness predicts citizens’ trust in government as it is mediated through social security satisfaction and life satisfaction. Previous studies mostly discussed the correlations among government performance, public service satisfaction, social fairness, and trust in government [5,6,17]. Further empirical studies are needed to explore the correlations between social security fairness and trust in government. In this study, we used data from the 2019 Chinese Social Survey (CSS) to explore the serial mediation effects of social security satisfaction and life satisfaction on the association between social security fairness and trust in government.

## 2. Literature Review and Research Hypothesis

### 2.1. Trust in Government

Citizens’ confidence in central and local government constitutes their trust in governments [3,5]. Citizens with a high level of trust in government are more willing to comply with government policies, respond to the government’s call, and participate in public affairs [19,20]. When citizens lose confidence in their government, they become reluctant to cooperate with the government [19,21], leading to increased costs and difficulty of governance and potentially causing the government to fall into the “Tacitus trap” [22]. Accordingly, it is important to investigate factors that might affect trust in government and help improve citizens’ confidence.

Trust in government has been a popular topic in political science research [23]. Institutional theories and cultural theories have provided completely different perspectives to explain the origin and development of trust in government [24]. Institutional theories hold that trust in government is politically endogenous [24]. Government performance mainly determines citizens’ trust in government, as based on a rational evaluation [24,25]. Trust fluctuates with fluctuations in a government’s economic and public service performance [4,5,25]. Cultural theories hold that trust in government is exogenous [24,25], originating from factors such as traditional culture, values, social capital, and individual experience [24,25]. Institutional theories and cultural theories are not mutually exclusive but complementary, with both considered the main theories explaining the origin of trust in government.

Supporters of institutional theories and cultural theories have investigated various factors of trust in government from different perspectives [5,11,17,18]. However, institutional theories ignore that social security fairness is an important basis for citizens to evaluate social security performance, whereas cultural theories neglect the effects of psychological feelings related to social security fairness on trust in government. Therefore, further empirical research is needed to investigate the associated mechanisms between social security fairness and trust in government, which could provide theoretical support for improving the level of citizens’ trust in government.

### 2.2. Social Security Fairness and Trust in Government

Social security fairness refers to the fairness of the process and results of social security services, which involves the fairness of multiple social security systems, such as elder security, public health security, and employment security [26]. The fairness theory proposed by Adams [27] suggests that people not only pay attention to the absolute value of the reward they received but also take note of its relative value to other rewards they or others have received. If people consider the rewards fair, they work more actively, thereby reducing workplace deviance [28]. Specifically, people’s perception of fairness affects their subsequent attitudes and behaviors [29]. Extending this concept to the study of trust in government, we examined the role of citizens’ attitudes toward their government in using social security services. If the social security services provided are perceived as fair and reasonable, the citizens are more likely to have higher levels of trust in their government.

Previous studies show that citizens have a strong dislike for the lack of fairness and equality [30,31]. The unfairness of public service resources and policy implementation can lead to their expectations falling short, thus damaging their trust in government [6,32]. Zmerli and Castillo [14] found that both income inequality and distributive unfairness are negatively associated with trust in government. Marien and Werner [7] also discovered that citizens who consider authorities to treat them fairly have greater trust in political institutions. Lee [6] confirmed that social fairness is positively correlated to trust in government.

On the basis of this evidence, we formulated a hypothesis about the relationship between social security fairness and trust in government.

**Hypothesis** **1** **(H1)**. *Social security fairness positively predicts citizens’ trust in government.*

### 2.3. The Mediator of Social Security Satisfaction

Social security satisfaction is defined as the overall satisfaction with various security systems. Expectancy disconfirmation theory holds that if the actual results exceed expectations, positive disconfirmation occurs and satisfaction emerges. If the actual results are lower than expected, negative disconfirmation occurs, leading to decreased satisfaction and complaints [33,34]. The fairness preference theory holds that human beings are born with a preference to pursue fairness [30]. Accordingly, citizens would have great expectations regarding social security fairness. When the perceived fairness in social security reaches or exceeds their expectations, citizens would evaluate social security services more positively, indicating greater social security satisfaction. On the contrary, when citizens believe that social security is unfair, negative disconfirmation, disappointment, and dissatisfaction with social security services will occur.

Several scholars have claimed that citizens’ satisfaction is closely correlated to trust in government. Welch et al. [10] confirmed that citizens’ satisfaction with e-government is positively associated with trust in government. Zhao and Hu [8] found that, compared with citizens who are unsatisfied with the quality of public service, satisfied citizens have greater trust in their government. Beeri et al. [9] found that citizens’ satisfaction with government is associated with trust in local government. Better quality of public services is associated with greater citizen satisfaction, as well as greater confidence in government [9,35]. Accordingly, it can be speculated that social security satisfaction affects trust in government.

On the basis of the above findings, we propose a hypothesis regarding social security fairness, social security satisfaction, and trust in government.

**Hypothesis** **2** **(H2).** *Social security satisfaction mediates the relationship between social security fairness and trust in government.*

### 2.4. The Mediator of Life Satisfaction

Life satisfaction is an individual’s overall subjective evaluation of their quality of life [36]. Research supports that subjective relative deprivation is a negative emotional experience, e.g., loss, dissatisfaction, and anger toward unfairness, which leads to a decline in an individual’s life satisfaction and happiness [37]. Liu and Pan [38] found that Chinese rural-to-urban migrant workers’ subjective relative deprivation is negatively associated with life satisfaction. Perception of unfairness is an indicator of relative deprivation [39]. Thus, social security unfairness may result in relative deprivation, negatively affecting life satisfaction. A previous study found that perceptions of social fairness and personal life satisfaction are highly correlated in EU countries [36]. Wang and Li [40] revealed that Wenchuan earthquake survivors who believed the government relief policy to be fair had a greater life satisfaction compared to those who did not. Sun and Xiao [13] confirmed that social security fairness significantly correlated with citizens’ life satisfaction.

Institutional theories hold that citizens’ life satisfaction is related to the government’s performance and is one of the institutional factors affecting trust in government [41]. On the basis of data from the four waves of the World Values Survey (WVS), Helliwell [42] found a positive linear relationship between life satisfaction and citizens’ evaluation of government. In general, the government’s actions affect citizens’ life satisfaction, which is highly correlated with trust in the government. Kong [18] confirmed that both competence-based trust in government and goodwill-based trust in government are positively related to citizens’ life satisfaction. Therefore, we propose a relationship linking social security fairness, life satisfaction, and trust in government.

**Hypothesis** **3** **(H3).** *Life satisfaction mediates the relationship between social security fairness and trust in government.*

### 2.5. The Serial Mediation Effects of Social Security Satisfaction and Life Satisfaction

Bottom-up and top-down theories are two approaches used to explain life satisfaction [43,44]. Top-down theories consider personality traits to be the main predictors of life satisfaction [44]. Bottom-up theories hold that life satisfaction is a function of satisfaction in all subareas of life, such as family, leisure, and work, and that a person’s satisfaction with all areas of life mainly determines their personal life satisfaction [45]. Lachmann et al. [46] discovered that personality variables contribute much less to the prediction of overall life satisfaction compared to such life satisfaction variables as work, family, and leisure. They concluded that their results support the bottom-up theories that life satisfaction in various areas of life (e.g., family, work) has a higher impact on overall life satisfaction than top-down variables of demographic and personality variables. Since social security is an essential aspect of daily life, we suggest that social security satisfaction should be highly correlated with citizens’ life satisfaction. Thus, we propose a serial two-mediator model describing social security fairness, social security satisfaction, life satisfaction, and trust in government.

**Hypothesis** **4** **(H4).** *Social security satisfaction and life satisfaction sequentially mediate the relationship between social security fairness and trust in government.*

## 3. Materials and Methods

### 3.1. Data and Sample

The data for this study came from the 2019 CSS, a nationally representative survey conducted by the Institute of Sociology at the Chinese Academy of Social Sciences. CSS performed a structured questionnaire administered in household interviews via the probability sampling method, covering 31 provinces of China, including 151 districts and 604 villages. Since 2005, it has used a biennial and continuous survey which involves covering between 7000 and 10,000 families on issues such as family and social life and social attitudes (for more information, please visit: http://css.cssn.cn/css_sy/ accessed on 7 April 2022).

The 2019 CSS collected 10,283 valid questionnaires; adults aged 18 and above were asked to respond to the survey questions. Respondents participated in the survey voluntarily and anonymously. Through processing the original data, the samples with missing values for the variables involved in the study were eliminated. The final sample included 7403 participants (44.99% males, 55.01% females). The participants’ mean age was 44.22 years old; 57.13% were educated below the senior high-school level, while 42.87% had an education at the senior high-school level or above. In addition, 59.34% were from urban areas, while 40.66% were from rural areas.

Because the 2019 CSS data were participants’ subjective self-reported answers, statistical measures were used to detect the presence of common method bias in the data [47]. The results of Harman’s single-factor test showed that the initial four factors extracted had eigenvalues greater than 1.0, and the first factor accounted for 36.90% of the total variance, which is less than the critical value of 40% [48], suggesting that our data had no serious common method bias.

### 3.2. Measures

#### 3.2.1. Criterion Variable

The criterion variable in this study was trust in government. In previous studies, researchers have measured overall trust in government as a function of participants’ trust in various hierarchies of the government, such as the central government and local government [5,49]. The 2019 CSS asked participants about their level of trust in central government, county government, and township government. The answers ranged from “no trust at all (1)” to “a great deal of trust (5)”. We used the average value of participants’ trust in central, county, and township governments as the level of overall trust in government, with higher scores reflecting greater levels of overall trust in government. Cronbach’s α coefficient for overall trust in government was 0.744. The KMO value was 0.566 (>0.5), and Bartlett’s test was significant (*p* < 0.001), indicating that the three items (trust at each level of government) were suitable for factor analysis [50]. The results of the principal component analysis (PCA) showed that one factor with an eigenvalue greater than 1.0 was retained, and it accounted for 67.244% of the total variance. Additionally, the factor loadings of the three items were 0.620, 0.922, and 0.885, respectively. The results of the confirmatory factor analysis (CFA) showed that the construct reliability (CR) of the three items was 0.784, and the average variance extracted (AVE) was 0.589, indicating that the scale had acceptable convergent validity.

#### 3.2.2. Predictor Variable

The predictor variable in this study was social security fairness. Social security is a general term used to refer to various social measures. In this study, social security fairness mainly refers to fairness in terms of public health, employment, and elder security. We measured social security fairness by asking participants the following question: “What do you think of the fairness of the following aspects in current social life: (a) public health, (b) work and employment opportunities, and (c) social security benefits such as elder security?” Respondents answered the question using a five-point rating scale: “very unfair (1)”, “generally unfair (2)”, “neither unfair nor fair (3)”, “generally fair (4)”, and “very fair (5)”. We used the average scores relating to public health fairness, employment fairness, and elder security fairness to represent the level of social security fairness. Higher scores indicated better social security fairness. Cronbach’s α coefficient for social security fairness was 0.659. The KMO value was 0.655 (>0.5), and Bartlett’s test was significant (*p* < 0.001), indicating that the three items were suitable for factor analysis. The results of the PCA showed that one factor was extracted which accounted for 59.453% of the total variance. Additionally, the factor loadings of the three items were 0.787, 0.744, and 0.781, respectively. The CFA results indicated that the AVE value was 0.394, and the CR value was 0.667.

#### 3.2.3. Mediator Variables

The first mediator variable in this study was social security satisfaction. Social security satisfaction was measured as overall satisfaction using three social security items: public health security, elder security, and employment security. To measure social security satisfaction, participants were instructed to “Please use a score of 1–10 to express your evaluation of the following social security items provided by the government to the people, where 1 means very dissatisfied and 10 means very satisfied: (a) public health security, (b) elder security, and (c) employment security.” In keeping with the 5-point rating scale used above, we converted the 10-point rating scale to a 5-point rating scale, whereby we coded scores of 1 and 2 as “1” and scores of 9 and 10 as “5”. A score of “1” meant “very dissatisfied”, while a score of “5” meant very satisfied. We took the average satisfaction with the three aspects as the index to measure the level of social security satisfaction. Cronbach’s α coefficient for social security satisfaction was 0.837. The KMO value was 0.713 (>0.5), and Bartlett’s test was significant (*p* < 0.001). The results of PCA showed that one factor was extracted which accounted for 75.457% of the total variance. The factor loadings of the three items were 0.888, 0.883, and 0.833, respectively. The CFA suggested that the AVE value was 0.387, and the CR value was 0.749.

The second mediator variable in this study was life satisfaction. Life satisfaction was measured as a function of the participants’ satisfaction with family relationships, family economic status, education level, leisure, and social life. We converted the 10-point rating scale to a 5-point rating scale, ranging from “very dissatisfied (1)” to “very satisfied (5)”. The average level of satisfaction with the five items was used to indicate the level of life satisfaction. Higher scores indicated that participants had greater satisfaction with their lives. Cronbach’s α coefficient for life satisfaction was 0.741. The KMO value was 0.756 (>0.5), and Bartlett’s test was significant (*p* < 0.001). The results of the PCA indicated that one factor was extracted which accounted for 49.946% of the total variance. The factor loadings of 5 items ranged from 0.491 to 0.811. The CFA suggested that the measurement of life satisfaction had acceptable convergent validity (AVE = 0.637 and CR = 0.843).

#### 3.2.4. Control Variables

We included gender (1 = male and 0 = female), age, education level (1 = senior high school or above and 0 = below senior high school), marital status (1 = married and 0 = not married or divorced), political status (1 = member of the Communist Party of China and 0 = others), region (1 = urban and 0 = rural), Internet use (1 = yes and 0 = no), and location (1 = in the east or west and 0 = others) in the model as control variables.

### 3.3. Statistical Analysis

We used SPSS 24.0 and Process 2.16 to conduct the statistical analyses. We employed descriptive statistics to examine the overall characteristics of the criterion and predictor variables. Correlation coefficients were computed to examine the strength of linear relationships among social security fairness, social security satisfaction, life satisfaction, and trust in government. Model 6 in Process 2.16 was used to test the serial mediation effects of social security satisfaction and life satisfaction on the relationship between social security fairness and trust in government at the central, county, and township levels.

## 4. Results

### 4.1. Descriptive Statistics and Correlation Analysis

Table 1 shows descriptive statistics and correlation coefficients. The average score for Chinese citizens’ overall trust in their government was 3.910. The central government enjoyed a higher level of trust than the county and township governments (M = 4.492, M = 3.745, and M = 3.494, respectively). Paired sample *t*-tests showed the three means differed significantly: (a) the mean difference between trust in central and county governments was 0.744 (*t* = 56.975, *df* = 7402, *p* < 0.001, medium Cohen’s *d* = 0.662), (b) the mean difference between trust in central and township governments was 0.998 (*t* = 65.466, *df* = 7402, *p* < 0.001, medium Cohen’s *d* = 0.761), and (c) the mean difference between trust in county and township governments was 0.251 (*t* = 25.905, *df* = 7402, *p* < 0.001, small Cohen’s *d* = 0.301). The average score of social security fairness across all levels was 3.491. Scores concerning citizens’ satisfaction with social security and life were also at a similar level (M = 3.453 and M = 3.471, respectively).

The correlation analysis showed that social security fairness was positively associated with overall trust in government (*r* = 0.401, *p* < 0.001). Social security fairness was significantly associated with trust in central, county, and township governments (*r* = 0.189, *p* < 0.001; *r* = 0.375, *p* < 0.001 and *r* = 0.387, *p* < 0.001, respectively). Social security satisfaction and life satisfaction were significantly positively associated with overall trust in government (*r* = 0.375, *p* < 0.001 and *r* = 0.259, *p* < 0.001, respectively). Social security satisfaction was also significantly positively associated with life satisfaction (*r* = 0.441, *p* < 0.001). Correlations among social security fairness, social security satisfaction, life satisfaction, and trust in government were all significant. We also found that social security fairness, social security satisfaction, and life satisfaction had the weakest correlations with trust in central government and the strongest correlations with trust in township government. The correlations between trust and other variables were higher for lower levels of government. Considering that correlations were significant among the variables, we performed several mediation analyses.

### 4.2. The Serial Mediation Effects of Social Security Satisfaction and Life Satisfaction

**Figure 1:**
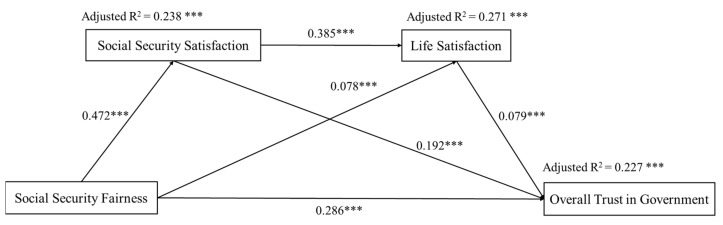
The serial mediator model of social security fairness, social security satisfaction, life satisfaction, and overall trust in government after adding the control variables (*n* = 7403). Standardized regression coefficients are shown next to the arrows. Adjusted R^2^ is shown above the explained variable. *** *p* < 0.001.

We used Amos software to analyze the overall fit of the tested models before path analysis. The results presented acceptable model fit indices (CFI = 0.946, TLI = 0.931, RMSEA = 0.060, SRMA = 0.034, and chi-square/*df* = 27.8). We used the bootstrap sampling method to test the serial mediation effect through Model 6 in the Process 2.16 plug-in of the SPSS macro program. The sample size was set to 5000, and the confidence level was 95%. Mediation analyses included the following control variables: gender, age, education level, marital status, political status, region, Internet use, and location. Figure 1 shows the results of the path analysis. The proposed model explained 23.8% of the variance in social security satisfaction (*p* < 0.001), 27.1% of the variance in life satisfaction (*p* < 0.001), and 22.7% of the variance in overall trust in government (*p* < 0.001). The results demonstrated that social security fairness had a positive and statistically significant direct effect on overall trust in government (β = 0.286, *p* < 0.001). The path coefficient between social security satisfaction and social security fairness was 0.472 (*p* < 0.001), indicating that social security fairness significantly positively predicted social security satisfaction. The path coefficient between overall trust in government and social security satisfaction was 0.192 (*p* < 0.001), showing that social security satisfaction significantly partially mediated the relationship between social security fairness and overall trust in government (β = 0.091, *p* < 0.001). In addition, the 95% confidence intervals of bootstrapping with a sample size of 5000 were 0.077 and 0.104, excluding 0.

The path coefficient between social security fairness and life satisfaction was 0.078 (*p* < 0.001), indicating that social security fairness significantly positively predicted life satisfaction. The path coefficient between life satisfaction and overall trust in government was 0.079 (*p* < 0.001). Life satisfaction partially mediated the association between social security fairness and overall trust in government (β = 0.006, *p* < 0.001). In addition, the 95% confidence intervals of bootstrapping with a sample size of 5000 were 0.004 and 0.010, excluding 0.

The path coefficient between social security satisfaction and life satisfaction was 0.385 (*p* < 0.001), indicating that life satisfaction was highly correlated with social security satisfaction. The results revealed that the serial mediation effects of social security satisfaction and life satisfaction between social security fairness and overall trust in government were significant (β = 0.014, *p* < 0.001). The 95% confidence intervals of bootstrapping with a sample size of 5000 were 0.010 and 0.019, excluding 0. Therefore, all path coefficients in the model reached the level of statistical significance (*p* < 0.001). Social security fairness indirectly partially predicted overall trust in government through social security satisfaction, life satisfaction, and the serial mediation of social security satisfaction and life satisfaction.

**Figure 2:**
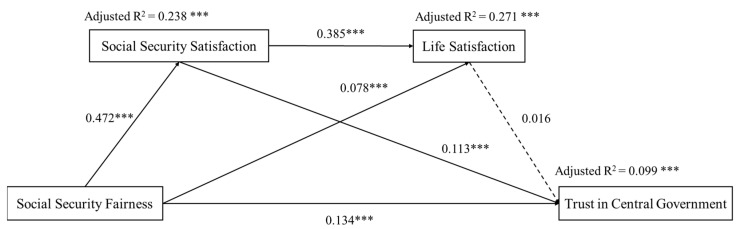
The serial mediator model of social security fairness, social security satisfaction, life satisfaction, and trust in central government after adding the control variables (*n* = 7403). Standardized regression coefficients are marked next to the arrows. Adjusted R^2^ is marked above the explained variable. *** *p* < 0.001.

We further examined the serial mediation effects that social security satisfaction and life satisfaction had on the relationship between social security fairness and trust in government at the central, county, and township levels. Figure 2 presents the results of the path analysis between social security fairness and trust in central government. After adding control variables, the path coefficient between social security fairness and trust in central government was 0.134 (*p* < 0.001), indicating that social security fairness directly and positively predicted trust in central government. The path coefficient between social security satisfaction and trust in central government was 0.113 (*p* < 0.001), showing that social security satisfaction partially mediated the relationship between social security fairness and trust in central government (β = 0.054, 95% CIs: 0.039, 0.067). Meanwhile, the path coefficient between life satisfaction and trust in central government was 0.016 (*p* > 0.05), indicating that the prediction of trust in central government using life satisfaction was not significant. Life satisfaction was not a significant mediator in the relationship between social security fairness and trust in central government. The results reveal that social security fairness cannot significantly and indirectly predict trust in central government through life satisfaction (95% CIs: −0.001, 0.035) and the serial mediation of social security satisfaction and life satisfaction (95% CIs: −0.002, 0.008). In addition, the serial model explained the change in trust in central government by 9.9% (*p* < 0.001).

**Figure 3:**
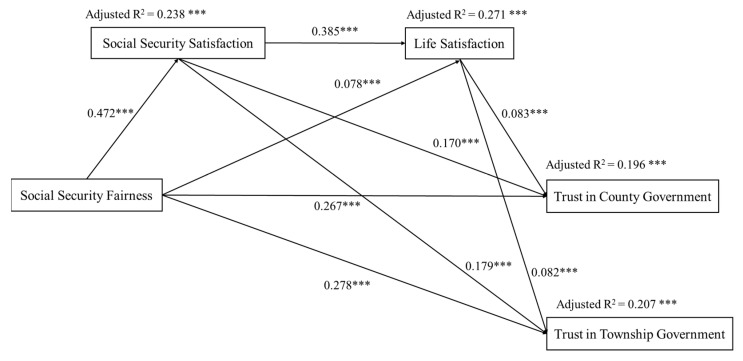
The serial mediator model of social security fairness, social security satisfaction, life satisfaction, and trust in local (county and township) government after adding the control variables (*n* = 7403). Standardized regression coefficients are shown next to the arrows. Adjusted R^2^ is shown above the explained variable. *** *p* < 0.001.

Figure 3 shows the results of the path analysis between social security fairness and trust in local government at the county and township levels. The serial mediation model explained 19.6% of the variance in trust in county government (*p* < 0.001). The path coefficients of social security fairness, social security satisfaction, and life satisfaction on trust in county government were 0.267 (*p* < 0.001), 0.170 (*p* < 0.001), and 0.083 (*p* < 0.001), respectively. Social security fairness indirectly predicted trust in county government through social security satisfaction, life satisfaction, and their serial mediation were 0.080 (95% CIs: 0.067, 0.094), 0.006 (95% CIs: 0.004, 0.010), and 0.015 (95% CIs: 0.010, 0.020), respectively.

The results of the path analysis between social security fairness and trust in township government show that the serial mediation model explained 20.7% of the variance in trust in township government (*p* < 0.001). The path coefficients from social security fairness, social security satisfaction, and life satisfaction to trust in township government were 0.278 (*p* < 0.001), 0.179 (*p* < 0.001), and 0.082 (*p* < 0.001), respectively. Social security fairness significantly and directly predicted trust in township government (β = 0.278, *p* < 0.001). Social security fairness and trust in township government were related through social security satisfaction (β = 0.085, 95% CIs: 0.072, 0.099), life satisfaction (β = 0.006, 95% CIs: 0.004, 0.010), and their serial mediation (β = 0.015, 95% CIs: 0.010, 0.020), respectively.

In addition, the regression results concerning the control variables revealed some demographic factors that predicted overall trust in government. Since a large sample size can influence the statistical significance of results, *p* = 0.001 was used to evaluate significance. Citizens’ age (β = 0.065, *p* < 0.001), education level (β = 0.075, *p* < 0.001), and political status (β = 0.062, *p* < 0.001) were significantly associated with their overall trust in government. The path coefficients from gender (β = −0.008, *p* > 0.001), marital status (β = −0.012, *p* > 0.001), region (β = −0.03, *p* > 0.001), Internet use (β = −0.03, *p* > 0.001), living in eastern China (β = 0.023, *p* > 0.001), or living in western China (β = −0.007, *p* > 0.001) to overall trust in government were not significant at the 0.001 level. Citizens who are older, have a higher education level, and are members of the Communist Party of China have a higher level of overall trust in government. We can also see that social security fairness is capable of significantly and positively predicting trust in central government (β = 0.134, *p* < 0.001), trust in county government (β = 0.267, *p* < 0.001), and trust in township government (β = 0.278, *p* < 0.001). In examining the adjusted R^2^ changes, the serial mediation model appeared to have a higher explanatory power to trust in county (adjusted R^2^ = 0.196, *p* < 0.001) and township (adjusted R^2^ = 0.207, *p* < 0.001) governments than in central government (adjusted R^2^ = 0.099, *p* < 0.001). The results illustrate the fact that the positive prediction of trust in government via social security fairness was better for lower levels of the government than for higher levels.

## 5. Discussion

The results of the descriptive statistical analysis and paired-samples *t*-tests showed that Chinese citizens’ trust in central government was significantly higher than in county and township governments. The effect sizes showed that the trust gap between the central and county and township governments was medium, but the trust gap between the latter two governments was small. This is consistent with the results of previous studies [51,52]. The hierarchical trust in government may be due to Chinese citizens’ inclination of regarding the central government as performing better than local governments [53]. The trust gap between the central and local governments in part reflected “the gap between central rhetoric and local practice” [54]. Chinese citizens’ social security fairness, social security satisfaction, and life satisfaction were at an average level, which may be caused by the government’s failure to meet the citizens’ demand for social security services.

The results indicated that social security fairness positively predicted trust in government, and the positive prediction of trust via social security fairness in the lower-level government was better than in higher-level government. Previous research has shown that maintaining social fairness is the government’s inherent duty and that social fairness is closely related to trust in government [6]. Social policy fairness, distributive fairness, and the fairness of the service delivery processes have been confirmed to positively predict citizens’ trust in government [7,14,40,55]. Our findings were consistent with previous results. However, we further discovered that the prediction of trust in local government (county and township government) using social security fairness was stronger compared to that of trust in central government. China’s governance system may explain this interesting finding. The Chinese governance system’s characteristics can be summarized as “vertically decentralized authoritarianism”; the central government governs the Chinese officials, while the local government governs the people [56]. The low-level governments execute more social security services, and the citizens have more contact with the low-level governments in the process of receiving social security services. Citizens interact more frequently with low-level governments. Therefore, the role of social security fairness in improving trust in low-level governments may be more obvious than in high-level governments. A previous study also found social fairness had a stronger effect on trust in local government compared to trust in central government [57].

The results showed that social security satisfaction partially mediated the relationship between social security fairness and overall trust in government including at the central, county, and township levels of government. Previous studies have demonstrated that the fairness of the service delivery process and citizens’ satisfaction with the quality of public services are highly associated with trust in government [8,58]. Our results are consistent with previous studies. If citizens perceive the process and outcome of social security service delivery as unfair, their satisfaction with social security will be significantly reduced, leading to complaints and the loss of trust in their government.

Figure 3 illustrates that social security fairness indirectly and partially predicted trust in government at the county and township levels through life satisfaction. Prior research has shown that social policy fairness positively predicts citizens’ life satisfaction [13,40]. This finding is consistent with previous studies. It is worth noting that life satisfaction did not have a statistically significant association with trust in central government. Life satisfaction was not a significant mediator in the relationship between social security fairness and trust in central government. In China, the central government is responsible for the formulation of policies, while local governments are responsible for the implementation of these policies. The governance system of “vertically decentralized authoritarianism” makes the county and township governments the main service providers in China. Thus, the quality of citizens’ life is more closely determined by the actions of the county and township governments than by the central government. Prior research has shown that improvements in family finances significantly increased citizens’ trust in county and township governments, but not in high-level governments [59]. Li found that citizens with lower life satisfaction had lower trust in government and even lower trust in local government, which was directly related to their perceptions of quality of life [60]. Therefore, improvements in life satisfaction can help increase trust in county and township governments.

Our results indicated that social security fairness indirectly positively predicted overall trust in government, county, and township governments through the serial mediation of social security fairness and life satisfaction. Zhou et al. [61] demonstrated that social security satisfaction significantly and positively predicted citizens’ life satisfaction. Since social security services affect all aspects of a citizen’s life, their dissatisfaction with social security may have a negative spillover effect that may negatively impact life satisfaction and trust in county and township governments. Therefore, formulating social policy to safeguard social security fairness is important for promoting trust in county and township governments.

## 6. Conclusions

We used a nationally representative survey to examine the mediation effects of social security satisfaction and life satisfaction on the association between social security fairness and trust in government. We found in 2019 that although the Chinese government enjoyed high levels of trust, there was stronger trust in the central than in the local governments. Our results suggest the need to improve social security fairness because it is likely to lead to higher levels of social security satisfaction, life satisfaction, and trust in government.

Trust was better predicted via social security at county and township levels than at the central government level. Furthermore, social security fairness indirectly and positively predicted trust in local government at the county and township levels through social security satisfaction and life satisfaction. Social security fairness only indirectly predicted trust in central government through social security satisfaction, and the prediction of trust in central government via life satisfaction was not significant. Therefore, improving social security fairness can help narrow the trust gap between the Chinese local and central governments. During the current pandemic, administering social security benefits in a fair manner is very important for ensuring that citizens’ needs are met adequately [62,63]. The Chinese government should strive to promote fairness in the distribution of social security to improve trust by building service-oriented local and township agencies.

Several limitations of this study need to be considered in the interpretation of the results. First, the CSS was a cross-sectional investigation, which made it impossible for us to determine the causal relationships between variables. We hope future studies will examine the possible causal relationship among variables. Second, the AVE values of scales measuring social security fairness and social security satisfaction were below 0.4, indicating that the convergent validity of the two scales was not ideal. We simply measured fairness and satisfaction in social security services from three aspects (public health, employment, and elder security). Future studies that examine the roles of fairness and satisfaction in determining trust in government should include other dimensions of social security services (e.g., housing security and minimum living security). Third, our results were specific to China, and it will be interesting to see if the same trends can be found in other countries despite differences in the political systems. Fourth, the data we used came from before the outbreak of the COVID-19, and therefore our results were not generalizable to the pandemic times. Future studies can compare the effects of social security fairness on trust in government before and after the pandemic. A longitudinal and multinational design is needed in pandemic times that examines the multiple mediation effects of social security fairness, social security satisfaction, life satisfaction, and trust in government.

## Figures and Tables

**Table 1:** Descriptive statistics and correlations among the variables.

Variables	M	SD	1	2	3	4	5	6
1 Trust in central government	4.492	0.803						
2 Trust in county government	3.745	1.180	0.403 ***					
3 Trust in township government	3.494	1.300	0.294 ***	0.778 ***				
4 Overall trust in government	3.910	0.912	0.606 ***	0.919 ***	0.897 ***			
5 Social security fairness	3.491	0.887	0.189 ***	0.375 ***	0.387 ***	0.401 ***		
6 Social security satisfaction	3.453	1.067	0.194 ***	0.346 ***	0.357 ***	0.375 ***	0.475 ***	
7 Life satisfaction	3.471	0.810	0.110 ***	0.247 ***	0.252 ***	0.259 ***	0.253 ***	0.441 ***

*** *p* < 0.001.

## Data Availability

Publicly available datasets were analyzed in this study. These data can be found at: http://css.cssn.cn/css_sy/, accessed on 7 April 2022.
